# Metabolic and Inflammatory Markers of Fatty Liver As Associated Factors of Hepatic Metastatic Spread in Colorectal Cancer

**DOI:** 10.7759/cureus.109707

**Published:** 2026-05-26

**Authors:** Syed Umar Farooq, Yasir Khan, Muhammad Sameer Khan, Umair Ali, Zhou Li, Reena Kumari Sunil

**Affiliations:** 1 General Surgery, Southern Medical University, Guangzhou, CHN; 2 Publich Health and Preventive Medicine, Guangdong Pharmaceutical University, Guangzhou, CHN; 3 Neurology, Medical School of Yangtze University, Jingzhou, CHN; 4 Medicine, Medical School of Nanchang University, Nanchang, CHN; 5 General Surgery, Zhujiang Hospital of Southern Medical University, Guangzhou, CHN; 6 Medicine, Dr. Ziauddin University Hospital, Karachi, PAK

**Keywords:** colorectal cancer, fatty liver, hepatic metastasis, inflammatory biomarkers, insulin resistance

## Abstract

Background

Colorectal cancer is a leading cause of cancer-related morbidity and mortality worldwide, and the development of hepatic metastasis significantly worsens patient prognosis. The liver is the most common site of metastatic spread in colorectal cancer due to portal venous drainage. Increasing evidence suggests that metabolic disorders and fatty liver disease may influence tumour progression by altering the hepatic microenvironment. However, the significance of metabolic and inflammatory markers associated with fatty liver in determining hepatic metastasis remains inadequately explored.

Methods

This observational analytical study was conducted at the Dr. Ziauddin University Hospital (Karachi, PAK) over one year from February 4, 2025, to February 3, 2026. A total of 200 patients with histologically confirmed colorectal adenocarcinoma were included using consecutive sampling. Fatty liver was assessed by abdominal ultrasonography and graded according to standard criteria. Metabolic parameters, including fasting glucose, fasting insulin, homeostatic model assessment for insulin resistance (HOMA-IR), and lipid profile, were measured. Inflammatory biomarkers, including total leukocyte count, neutrophil-to-lymphocyte ratio, platelet-to-lymphocyte ratio, and high-sensitivity C-reactive protein (hs-CRP), were evaluated. Hepatic metastasis was determined using contrast-enhanced imaging. Logistic regression analysis was performed to identify factors associated with hepatic metastatic spread.

Results

Among the 200 patients included in the study, hepatic metastasis was identified in 62 (31.0%). Patients with hepatic metastasis had a significantly higher BMI, waist circumference, and prevalence of diabetes mellitus. Moderate-to-severe fatty liver was significantly associated with metastatic disease (p < 0.001). Metabolic markers, including fasting glucose, insulin levels, HOMA-IR, triglycerides, and liver enzymes, were significantly elevated in the metastatic group. Inflammatory markers such as the neutrophil-to-lymphocyte ratio and hs-CRP were also significantly higher. Multivariable logistic regression analysis revealed that moderate-to-severe fatty liver (adjusted OR = 2.18), HOMA-IR (adjusted OR = 1.31), triglycerides (adjusted OR = 1.01), hs-CRP (adjusted OR = 1.15), neutrophil-to-lymphocyte ratio (adjusted OR = 1.54), and advanced tumour stage (adjusted OR = 2.47) were associated with hepatic metastatic spread.

Conclusion

Metabolic dysfunction, fatty liver severity, and systemic inflammatory markers are significantly associated with hepatic metastasis in colorectal cancer. These parameters may serve as a potential association for identifying patients at increased risk of metastatic spread and may assist in improving risk stratification and surveillance strategies.

## Introduction

Colorectal cancer (CRC) is one of the most common malignancies worldwide and remains a major cause of cancer-related mortality [1]. According to the global cancer statistics of the International Agency for Research on Cancer, colorectal cancer ranks among the top three most frequently diagnosed cancers and is a leading cause of cancer deaths globally [2]. Despite advances in screening, surgical techniques, and systemic therapies, the prognosis of CRC patients is largely determined by the development of distant metastasis, particularly to the liver [3].

The liver is the most common site of metastasis in colorectal cancer because venous drainage from the colon and rectum occurs through the portal circulation directly to the liver. Approximately 20% to 30% of patients present with liver metastases at diagnosis, and nearly half of CRC patients develop hepatic metastasis during the course of the disease [4]. Hepatic metastatic spread significantly worsens prognosis and remains a major determinant of survival in colorectal cancer patients [5].

In recent years, increasing attention has been given to the role of metabolic disorders in cancer progression. Non‑alcoholic fatty liver disease (NAFLD), which represents the hepatic manifestation of metabolic syndrome, is characterised by excessive fat accumulation in hepatocytes and is strongly associated with obesity, insulin resistance, dyslipidaemia, and chronic low-grade inflammation [6]. It has become highly prevalent worldwide and is increasingly recognised not only as a liver disease but also as a systemic metabolic condition with significant oncologic implications [7].

Several biological mechanisms suggest that fatty liver may influence tumour metastasis to the liver. Hepatic steatosis is associated with an altered hepatic microenvironment, increased oxidative stress, inflammatory cytokine production, and immune dysregulation, which may promote tumour cell implantation and growth [8]. Experimental and clinical studies have suggested that fatty liver disease may increase susceptibility to colorectal and liver metastasis by creating a pro-inflammatory and pro-tumorigenic hepatic environment [9].

Metabolic abnormalities commonly associated with fatty liver, including insulin resistance, hyperglycemia, and dyslipidaemia, have also been implicated in cancer progression. Elevated insulin and insulin-like growth factor signalling may enhance tumour cell proliferation, angiogenesis, and metastatic potential [10]. Similarly, systemic inflammation reflected by biomarkers such as C-reactive protein (CRP), neutrophil-to-lymphocyte ratio, and platelet-to-lymphocyte ratio has been shown to be associated with poor outcomes and metastasis in colorectal cancer [11].

Although several studies have independently examined the roles of metabolic syndrome, fatty liver disease, or inflammatory markers in colorectal cancer progression, limited evidence is available regarding their combined association with hepatic metastatic spread. Understanding these associations may help identify patients at higher risk for metastasis and improve risk stratification and surveillance strategies. The objective of this study was to evaluate the factors associated with the metabolic and inflammatory markers related to fatty liver in determining the risk of hepatic metastasis among patients with colorectal cancer.

## Materials and methods

Study design and setting

This hospital-based cross-sectional analytical study was conducted at the Department of Gastroenterology and Hepatobiliary Surgery in Dr. Ziauddin University Hospital (Karachi, PAK). The study was carried out over one year from February 4, 2025, to February 3, 2026. The research protocol was developed and reported in accordance with the Strengthening the Reporting of Observational Studies in Epidemiology (STROBE) guidelines for observational studies [12]. Ethical approval was obtained from the Ethical Review Committee of Ziauddin University (approval No. ZIUD-ERC-2025/PA-159, dated February 3, 2025). Written informed consent was obtained from all participants prior to enrolment. All procedures were conducted in accordance with the principles outlined in the Declaration of Helsinki.

Study population and sampling technique

The study population comprised adult patients presenting with histologically confirmed colorectal adenocarcinoma at the study centre during the study period. Participants were recruited using a consecutive non-probability sampling technique, whereby all eligible patients were included until the required sample size was achieved.

Sample size calculation

**Figure 1:**
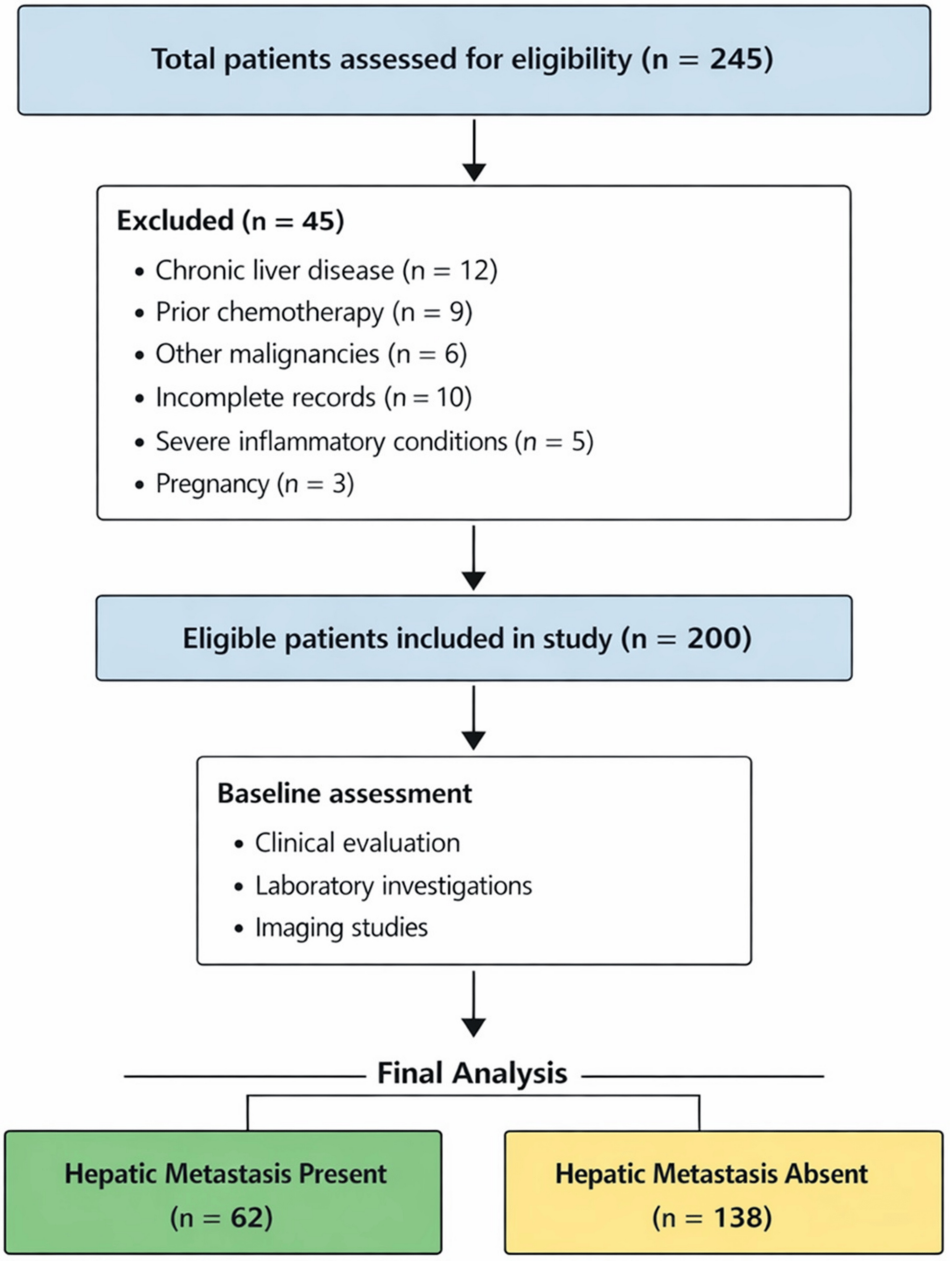
Flowchart illustrating patient selection and cohort formation in the study

The sample size was determined using standard formulas for analytical observational studies comparing proportions between two groups. Based on prior literature indicating an approximate 30% prevalence of hepatic metastasis in colorectal cancer patients, with a confidence level of 95%, statistical power of 80%, and an anticipated odds ratio of 2.0, the minimum sample size was calculated to be 168 participants. To account for potential missing data and dropouts, the sample size was increased by 15%, resulting in a final sample size of 200 patients (Figure 1).

Eligibility criteria

Patients aged 18 years or older with newly diagnosed, histopathologically confirmed colorectal adenocarcinoma who had undergone baseline imaging for staging and had complete clinical and laboratory records were included in the study. Patients were excluded if they had chronic liver diseases unrelated to metabolic fatty liver disease, including viral hepatitis B or C, autoimmune hepatitis, Wilson’s disease, primary biliary cholangitis, haemochromatosis, or alcoholic liver disease. Additional exclusions included prior treatment for colorectal cancer, administration of chemotherapy before baseline evaluation, presence of other primary malignancies, incomplete records, pregnancy, and severe systemic inflammatory conditions such as sepsis or autoimmune disorders.

Outcome assessment

The primary outcome of the study was the presence of hepatic metastasis. This was determined through radiological imaging using contrast-enhanced CT or MRI, interpreted by experienced radiologists based on standard diagnostic criteria for metastatic lesions. Radiological assessments were performed by experienced radiologists using standardised imaging protocols. The radiologists were blinded to the patients’ clinical details, laboratory findings, and metabolic and inflammatory marker status to minimise observer bias. The diagnosis of hepatic metastasis was based on characteristic imaging features observed on contrast-enhanced CT or MRI, consistent with established radiological criteria used in routine clinical practice. Histopathological confirmation was performed in selected cases where imaging findings were inconclusive or when clinically indicated.

Exposure variables and data collection

Baseline data collection included demographic characteristics, clinical history, anthropometric measurements, laboratory parameters, and imaging findings. The BMI was calculated using measured height and weight, while waist circumference was recorded using standard techniques. Blood samples were obtained after an overnight fast of at least eight hours. Fasting glucose levels were measured using enzymatic methods, and serum insulin levels were determined through chemiluminescent immunoassay. Insulin resistance was calculated using the homeostatic model assessment for insulin resistance (HOMA-IR). Lipid profiles, including total cholesterol, triglycerides, high-density lipoprotein cholesterol, and low-density lipoprotein cholesterol, were measured using enzymatic colorimetric methods. Liver enzymes, including alanine aminotransferase and aspartate aminotransferase, were assessed using standardised laboratory techniques. High-sensitivity C-reactive protein (hs-CRP) was measured using an immunoturbidimetric assay. Haematological parameters were obtained from an automated analyser, and inflammatory indices such as the neutrophil-to-lymphocyte ratio were calculated. Hepatic steatosis was assessed using ultrasonography and graded as mild, moderate, or severe based on established sonographic criteria.

Data quality assurance and missing data handling

Quality assurance measures included standardised laboratory procedures, independent review of imaging by two radiologists, and double data entry verification. Missing data were assessed for pattern and extent; complete case analysis was performed when missing values were less than 5%, while multiple imputation using chained equations was applied when missingness exceeded this threshold. 

Statistical analysis

All statistical analyses were conducted using R statistical software version 4.3.2 (The R Foundation for Statistical Computing, Vienna, AUT). Continuous variables were assessed for normality using the Shapiro-Wilk test and were presented as mean with standard deviation or median with interquartile range, as appropriate. Categorical variables were expressed as frequencies and percentages. Comparisons between patients with and without hepatic metastasis were performed using the independent sample t-test or Mann-Whitney U test for continuous variables and the chi-square test or Fisher’s exact test for categorical variables. Univariate logistic regression analysis was initially performed to identify potential factors associated with hepatic metastasis. Variables with a p-value less than 0.10 were subsequently entered into a multivariable logistic regression model to determine associated factors. Adjusted odds ratios with 95% confidence intervals were calculated, and a p-value less than 0.05 was considered statistically significant.

Multicollinearity among independent variables was assessed using the variance inflation factor (VIF), and all variables included in the multivariable model demonstrated VIF values <2, indicating no significant multicollinearity. The adequacy of the regression model was further ensured by maintaining an appropriate events-per-variable ratio, with 62 metastatic events and six predictors included in the final multivariable model, satisfying the recommended threshold of at least 10 events per variable.

## Results

The study included 200 patients with histologically confirmed colorectal cancer, of whom 62 (31.0%) had hepatic metastasis. Patients with hepatic metastasis were older, with a mean age of 58.6 ± 11.8 years compared to 54.6 ± 12.9 years in non-metastatic patients (p = 0.041). Although males constituted a higher proportion in both groups, the difference was not statistically significant (40 (64.5%) vs. 81 (58.7%), p = 0.447). Notably, patients with hepatic metastasis had significantly higher BMI (29.1 ± 4.3 vs. 26.8 ± 3.9 kg/m², p = 0.002) and waist circumference (101.7 ± 9.5 vs. 95.8 ± 8.7 cm, p < 0.001), suggesting a link between central obesity and liver metastasis. The prevalence of diabetes mellitus was significantly higher in metastatic patients (33 (53.2%) vs. 53 (36.2%), p = 0.028), while hypertension and smoking history did not differ significantly. Tumour location in the colon or rectum was comparable between groups, but advanced tumour stage (III-IV) was markedly higher in patients with hepatic metastasis (48 (77.4%) vs. 69 (50.0%), p < 0.001), reflecting the expected association of stage with metastatic potential (Table 1). 

**Table 1:** Baseline demographic and clinical characteristics of study participants (total n = 200) Categorical variables were analysed using the chi-square test, and continuous variables were analysed using the independent t-test.

Variable	Hepatic metastasis (n = 62)	No hepatic metastasis (n = 138)	p-value
Age (years), mean ± SD	58.6 ± 11.8	54.6 ± 12.9	0.041
Male, n (%)	40 (64.5)	81 (58.7)	0.447
Female, n (%)	22 (35.5)	57 (41.3)	-
BMI (kg/m²), mean ± SD	29.1 ± 4.3	26.8 ± 3.9	0.002
Waist circumference (cm), mean ± SD	101.7 ± 9.5	95.8 ± 8.7	<0.001
Diabetes mellitus, n (%)	33 (53.2)	53 (36.2)	0.028
Hypertension, n (%)	28 (45.2)	52 (37.7)	0.327
Smoking history, n (%)	21 (33.9)	38 (27.5)	0.366
Colon cancer location, n (%)	37 (59.7)	83 (60.1)	0.956
Rectal cancer location, n (%)	25 (40.3)	55 (39.9)	-
Advanced tumor stage (III-IV), n (%)	48 (77.4)	69 (50.0)	<0.001

Ultrasonography revealed that 112 (56.0%) participants had fatty liver disease. Moderate-to-severe steatosis was significantly more frequent in patients with hepatic metastasis (moderate: 16 (25.8%) vs. 14 (10.1%); severe: nine (14.5%) vs. six (4.3%)), while no fatty liver was more common in non-metastatic patients (71 (51.4%) vs. 17 (27.4%)). The overall association between fatty liver severity and hepatic metastasis was statistically significant (p < 0.001), indicating that higher grades of hepatic steatosis may predispose patients to metastatic liver involvement (Table 2).

**Table 2:** Ultrasonographic grading of fatty liver among study participants Fatty liver severity was classified as: none, mild, moderate, severe; the variables were analyzed using the chi-square test.

Fatty liver grade	Hepatic metastasis (n = 62)	No hepatic metastasis (n = 138)	p-value
No fatty liver	17 (27.4%)	71 (51.4%)	-
Mild steatosis	20 (32.3%)	47 (34.1%)	
Moderate steatosis	16 (25.8%)	14 (10.1%)	
Severe steatosis	9 (14.5%)	6 (4.3%)	
Overall association (chi-square)	-	-	<0.001

Patients with hepatic metastasis exhibited significantly higher fasting glucose (118.3 ± 26.7 vs. 104.2 ± 21.4 mg/dL, p < 0.001), fasting insulin (16.9 ± 6.4 vs. 12.8 ± 5.7 µIU/mL, p < 0.001), and HOMA-IR (4.96 ± 2.03 vs. 3.28 ± 1.66, p < 0.001), suggesting pronounced insulin resistance. Lipid abnormalities were also observed, with higher triglycerides (188.7 ± 56.2 vs. 153.8 ± 49.5 mg/dL, p < 0.001), higher total cholesterol (206.5 ± 34.9 vs. 192.4 ± 31.8 mg/dL, p = 0.009), and lower high-density lipoprotein (HDL) cholesterol (38.7 ± 7.4 vs. 43.2 ± 8.1 mg/dL, p = 0.001). Liver enzymes, alanine aminotransferase (ALT) (61.8 ± 25.4 vs. 44.7 ± 19.6 U/L, p < 0.001) and aspartate aminotransferase (AST) (54.2 ± 21.7 vs. 39.3 ± 16.8 U/L, p < 0.001), were significantly elevated in metastatic patients, reflecting hepatic metabolic stress and possible tumour infiltration (Table 3).

**Table 3:** Comparison of metabolic parameters between groups Independent t-tests were used for continuous variables. HOMA-IR: Homeostatic model assessment for insulin resistance; ALT: Alanine aminotransferase; AST: Aspartate aminotransferase; HDL: High-density lipoprotein

Parameter	Hepatic metastasis (n = 62)	No hepatic metastasis (n = 138)	p-value
Fasting glucose (mg/dL)	118.3 ± 26.7	104.2 ± 21.4	<0.001
Fasting insulin (µIU/mL)	16.9 ± 6.4	12.8 ± 5.7	<0.001
HOMA-IR	4.96 ± 2.03	3.28 ± 1.66	<0.001
Total cholesterol (mg/dL)	206.5 ± 34.9	192.4 ± 31.8	0.009
Triglycerides (mg/dL)	188.7 ± 56.2	153.8 ± 49.5	<0.001
HDL cholesterol (mg/dL)	38.7 ± 7.4	43.2 ± 8.1	0.001
Low-density lipoprotein (LDL) cholesterol (mg/dL)	133.5 ± 29.1	120.3 ± 27.8	0.004
ALT (U/L)	61.8 ± 25.4	44.7 ± 19.6	<0.001
AST (U/L)	54.2 ± 21.7	39.3 ± 16.8	<0.001

Inflammatory markers were significantly higher in patients with hepatic metastasis. Total leukocyte count (9.8 ± 2.6 vs. 7.9 ± 2.1 ×10⁹/L, p < 0.001), neutrophil percentage (68.4 ± 9.1 vs. 61.7 ± 8.6%, p < 0.001), platelet count (324 ± 89 vs. 287 ± 76 ×10⁹/L, p = 0.006), neutrophil-to-lymphocyte ratio (3.61 ± 1.42 vs. 2.31 ± 1.05, p < 0.001), platelet-to-lymphocyte ratio (182.5 ± 61.7 vs. 139.8 ± 52.4, p < 0.001), and hs-CRP (9.7 ± 4.6 vs. 5.3 ± 3.1 mg/L, p < 0.001) were all significantly elevated, indicating systemic inflammatory activation in patients with liver metastasis (Table 4).

**Table 4:** Inflammatory biomarkers among study participants Continuous variables were analysed using the independent t-test;  hs-CRP: High-sensitivity C-reactive protein

Parameter	Hepatic metastasis (n = 62)	No hepatic metastasis (n = 138)	p-value
Total leukocyte count (×10⁹/L)	9.8 ± 2.6	7.9 ± 2.1	<0.001
Neutrophils (%)	68.4 ± 9.1	61.7 ± 8.6	<0.001
Lymphocytes (%)	21.3 ± 6.7	27.4 ± 7.1	<0.001
Platelet count (×10⁹/L)	324 ± 89	287 ± 76	0.006
Neutrophil-to-lymphocyte ratio	3.61 ± 1.42	2.31 ± 1.05	<0.001
Platelet-to-lymphocyte ratio	182.5 ± 61.7	139.8 ± 52.4	<0.001
hs-CRP (mg/L)	9.7 ± 4.6	5.3 ± 3.1	<0.001

Univariate logistic regression revealed several significant factors associated with hepatic metastasis. Moderate-to-severe fatty liver had the highest odds ratio (OR = 2.74, 95% CI: 1.49-5.03, p = 0.001). Other significant factors included BMI (OR = 1.11, p = 0.002), diabetes mellitus (OR = 2.01, p = 0.024), HOMA-IR (OR = 1.42, p < 0.001), triglycerides (OR = 1.01, p = 0.003), ALT (OR = 1.02, p < 0.001), hs-CRP (OR = 1.19, p < 0.001), and neutrophil-to-lymphocyte ratio (OR = 1.67, p < 0.001), highlighting the combined contribution of metabolic dysfunction and systemic inflammation in promoting hepatic metastasis (Table 5).

**Table 5:** Univariate logistic regression analysis for the factors associated with hepatic metastasis A p-value < 0.05 was considered statistically significant. HOMA-IR: Homeostatic model assessment of insulin resistance; ALT: Alanine aminotransferase; hs-CRP: High-sensitivity C-reactive protein

Variable	Odds ratio (OR)	95% CI	p-value
BMI	1.11	1.04-1.19	0.002
Diabetes mellitus	2.01	1.09-3.70	0.024
HOMA-IR	1.42	1.21-1.66	<0.001
Triglycerides	1.01	1.00-1.02	0.003
ALT	1.02	1.01-1.03	<0.001
hs-CRP	1.19	1.10-1.30	<0.001
Neutrophil-lymphocyte ratio	1.67	1.34-2.07	<0.001
Moderate-to-severe fatty liver	2.74	1.49-5.03	0.001

After adjusting for confounders, moderate-to-severe fatty liver remained a significant associated factor of hepatic metastasis (adjusted OR = 2.18, 95% CI: 1.14-4.19, p = 0.019). Other independent factors included HOMA-IR (adjusted OR = 1.31, p = 0.002), triglycerides (adjusted OR = 1.01, p = 0.021), hs-CRP (adjusted OR = 1.15, p = 0.003), neutrophil-to-lymphocyte ratio (adjusted OR = 1.54, p < 0.001), and advanced tumour stage (adjusted OR = 2.47, p = 0.008). These results suggest that both metabolic and inflammatory parameters, alongside tumour characteristics, are independently associated with hepatic metastatic spread in colorectal cancer (Table 6).

**Table 6:** Multivariable logistic regression model for the factors associated with hepatic metastatic spread Multivariable logistic regression was performed using the enter method; a p-value < 0.05 was considered statistically significant; adjusted OR: Odds ratio adjusted for age, sex, tumor stage, diabetes, and obesity; HOMA-IR: Homeostatic model assessment of insulin resistance; ALT: Alanine aminotransferase; hs-CRP: High-sensitivity C-reactive protein

Variable	Adjusted OR	95% CI	p-value
Moderate-to-severe fatty liver	2.18	1.14-4.19	0.019
HOMA-IR	1.31	1.10-1.55	0.002
Triglycerides	1.01	1.00-1.02	0.021
hs-CRP	1.15	1.05-1.26	0.003
Neutrophil-lymphocyte ratio	1.54	1.21-1.95	<0.001
Advanced tumor stage	2.47	1.26-4.83	0.008

## Discussion

The present study investigated the role of metabolic and inflammatory markers linked with fatty liver disease as associated factors of hepatic metastatic spread in colorectal cancer. The findings demonstrate that metabolic dysregulation, hepatic steatosis, and systemic inflammatory responses are significantly associated with the occurrence of liver metastasis. Importantly, moderate-severe fatty liver, insulin resistance, elevated triglycerides, high hs-CRP, and increased neutrophil-to-lymphocyte ratio remained independent factors associated with hepatic metastatic spread after adjustment for confounders.

Age was significantly higher in patients with hepatic metastasis in the present study. Increasing age has been reported as an important factor influencing cancer progression and metastatic potential. Older individuals frequently demonstrate metabolic dysregulation, impaired immune surveillance, and cumulative genetic mutations, which may facilitate tumour dissemination. Similar observations have been reported by Chang et al., who reported that advanced age was associated with higher metastatic burden and poorer outcomes in colorectal cancer [13]. However, some studies have reported inconsistent associations, suggesting that tumour biology and stage may exert stronger influences than chronological age alone.

Sex distribution in our study did not significantly differ between groups. Although some epidemiological studies have suggested that males have a slightly higher incidence of colorectal cancer and metastasis, the association between sex and hepatic metastatic spread remains inconsistent [14]. Yu et al. reported that sex differences were not independently associated with metastatic risk when metabolic and tumour-related factors were adjusted for [15]. Our findings, therefore, align with evidence suggesting that metabolic and inflammatory parameters may play a more decisive role than sex alone.

The BMI and waist circumference were significantly higher among patients with hepatic metastasis. Obesity is increasingly recognised as a key driver of colorectal cancer progression. Excess visceral adiposity promotes chronic inflammation, insulin resistance, and altered adipokine signalling, all of which contribute to tumour growth and metastasis [16]. Researchers demonstrated that obesity-related metabolic abnormalities increase colorectal cancer aggressiveness through activation of insulin and insulin-like growth factor signalling pathways [17]. Similarly, epidemiological studies have shown that central obesity measured by waist circumference is more strongly associated with colorectal cancer metastasis than BMI alone [18]. Our findings, therefore, support the concept that visceral adiposity creates a tumour-promoting metabolic environment.

Diabetes mellitus was significantly more prevalent among patients with hepatic metastasis in this study. Diabetes has long been linked with colorectal cancer progression due to hyperinsulinemia and increased circulating insulin-like growth factor 1 levels. These factors stimulate cellular proliferation and inhibit apoptosis, thereby facilitating tumour spread. A large population-based cohort study conducted by Giovannucci et al. demonstrated that patients with diabetes had significantly higher risks of colorectal cancer incidence and mortality [10]. Our findings are consistent with these reports and further suggest that diabetes-associated metabolic alterations may also contribute specifically to hepatic metastasis.

Tumour stage was significantly associated with hepatic metastasis in our cohort, with a markedly higher proportion of advanced-stage disease among metastatic patients. This finding is expected because tumour invasion depth and nodal involvement are key determinants of metastatic spread. Previous studies have consistently demonstrated that stage III and IV colorectal cancers carry substantially higher risks of liver metastasis [4]. The present findings, therefore, reinforce the established relationship between advanced tumour stage and metastatic dissemination.

Fatty liver disease was highly prevalent in our study population and showed a strong association with hepatic metastasis. Moderate-to-severe steatosis was significantly more frequent in patients with metastatic disease. Emerging evidence suggests that fatty liver may create a favourable microenvironment for tumour cell implantation and growth. Hepatic steatosis alters liver sinusoidal architecture, promotes oxidative stress, and induces inflammatory cytokine production, thereby enhancing metastatic colonisation. Previous research also demonstrated that fatty liver significantly increases susceptibility to colorectal cancer liver metastasis by promoting hepatic inflammatory signalling [9]. Clinical studies have also supported this relationship, showing higher metastatic rates among patients with non-alcoholic fatty liver disease. Our results, therefore, contribute to the growing evidence that hepatic steatosis is not merely a metabolic condition but may actively influence metastatic tumour biology.

Metabolic biomarkers were also significantly altered in patients with hepatic metastasis. Fasting glucose, insulin levels, and HOMA-IR were all significantly elevated among metastatic patients, indicating increased insulin resistance. Insulin resistance promotes tumour progression through multiple mechanisms, including activation of PI3K/AKT signalling, increased insulin-like growth factor activity, and enhanced cellular proliferation. Research by Vegas-Suarez et al. demonstrated that insulin resistance associated with metabolic syndrome significantly increases the risk of colorectal cancer progression and metastasis [19]. Our findings showing HOMA-IR as an independent factor associated with hepatic metastasis further support this metabolic-oncologic link.

Dyslipidaemia was another important metabolic alteration observed in our study. Patients with hepatic metastasis demonstrated significantly higher triglycerides, higher low-density lipoprotein (LDL) cholesterol, and lower HDL cholesterol levels. Lipid abnormalities have been implicated in tumour metabolism and metastatic potential. Tumour cells rely heavily on lipid metabolism for energy production and membrane synthesis during rapid proliferation. A study by Kumar et al. highlighted the role of altered lipid metabolism in supporting cancer cell survival and metastasis [20]. Elevated triglyceride levels, which remained independently associated with metastasis in our analysis, may therefore reflect metabolic conditions that favour tumour progression.

Liver enzymes, including alanine aminotransferase and aspartate aminotransferase, were significantly higher in metastatic patients. These elevations likely reflect underlying hepatic injury associated with fatty liver as well as metastatic infiltration. Elevated aminotransferases have previously been reported as indicators of liver dysfunction in colorectal cancer patients with metastatic disease [21]. However, these markers are not specific for metastasis and should be interpreted alongside imaging and other biomarkers.

Inflammatory biomarkers were strongly associated with hepatic metastasis in the present study. Total leukocyte count, neutrophil percentage, platelet count, neutrophil-to-lymphocyte ratio, platelet-to-lymphocyte ratio, and hs-CRP were all significantly elevated among patients with metastatic disease. Chronic inflammation is a well-recognised hallmark of cancer progression [22]. Neutrophils promote tumour growth through the secretion of cytokines, growth factors, and proteases that facilitate tumour invasion and angiogenesis [23]. Conversely, lymphocytes play an important role in anti-tumour immunity [24]. Therefore, an elevated neutrophil-to-lymphocyte ratio reflects a shift toward a pro-tumour inflammatory environment.

Previous studies have confirmed the prognostic value of inflammatory indices in colorectal cancer. A meta-analysis by Li et al. demonstrated that an elevated neutrophil-to-lymphocyte ratio was associated with poorer survival and increased metastatic potential in colorectal cancer [25]. Similarly, an elevated platelet-to-lymphocyte ratio has been linked with tumour progression and metastasis due to platelet-mediated protection of circulating tumour cells [23]. Our findings showing a significantly higher neutrophil-to-lymphocyte ratio and platelet-to-lymphocyte ratio among metastatic patients, therefore, corroborate these previous observations.

High-sensitivity CRP was also significantly elevated in patients with hepatic metastasis and remained independently associated in multivariable analysis. C-reactive protein reflects systemic inflammatory activity and has been associated with poor outcomes in several malignancies. Research has demonstrated that elevated CRP levels are associated with increased recurrence and metastasis in colorectal cancer patients [26]. However, given the cross-sectional nature of the study, elevated CRP levels in our cohort may reflect underlying tumour burden or systemic inflammatory response secondary to existing metastasis rather than a causal role in metastatic progression.

Multivariable logistic regression analysis demonstrated that moderate-to-severe fatty liver, insulin resistance, hypertriglyceridemia, elevated hs-CRP, increased neutrophil-to-lymphocyte ratio, and advanced tumour stage were independently associated with hepatic metastatic spread. These findings suggest a potential link between metabolic dysregulation, systemic inflammation, and the presence of hepatic metastasis; however, due to the lack of temporal assessment, these variables should be interpreted as associated factors rather than true predictors. Fatty liver and insulin resistance may alter the hepatic microenvironment, while systemic inflammation may facilitate tumour cell survival and migration. Nevertheless, these mechanisms remain speculative in the context of this study design and require confirmation through longitudinal studies to establish causality and predictive value.

The use of ultrasonography for fatty liver assessment is operator-dependent and has limited sensitivity, particularly for mild steatosis, and inter-observer variability was not formally assessed. Although efforts were made to standardise imaging interpretation, radiologists were not formally blinded, which may introduce observer bias. Additionally, the cross-sectional design limits the ability to establish temporal relationships or causal inference. The single-centre nature of the study may also limit the generalisability of the findings to broader populations. These factors should be considered when interpreting the results.

## Conclusions

The findings of this study support the emerging concept that metabolic syndrome and chronic inflammation are important contributors to cancer progression. Early identification of patients with metabolic abnormalities and inflammatory activation may therefore help identify individuals at higher risk for hepatic metastasis. This could facilitate closer surveillance, early intervention, and targeted metabolic management strategies in colorectal cancer patients.
